# Pharmacists’ perspectives on medication therapy management training needs for chronic disease care in Indonesian primary healthcare centers: a qualitative study across 32 provinces

**DOI:** 10.1186/s12909-026-09722-6

**Published:** 2026-06-19

**Authors:** Farida Rendrayani, Sofa Dewi Alfian, Thang Nguyen, Irma Melyani Puspitasari

**Affiliations:** 1 Doctoral Program of Pharmacy, Faculty of Pharmacy, Universitas Padjadjaran, Sumedang, Indonesia; 2 Department of Pharmacology and Clinical Pharmacy, Faculty of Pharmacy, Universitas Padjadjaran, Sumedang, Indonesia; 3 Center of Excellence for Pharmaceutical Care Innovation, Universitas Padjadjaran, Sumedang, Indonesia; 4 Center for Health Technology Assessment, Universitas Padjadjaran, Sumedang, Indonesia; 5 Department of Pharmacology and Clinical Pharmacy, Faculty of Pharmacy, Can Tho University of Medicine and Pharmacy, Can Tho City, Vietnam

**Keywords:** Pharmacists, Primary Healthcare Centers, Medication Therapy Management, Chronic Disease, Clinical Pharmacy Training

## Abstract

**Background:**

Chronic diseases place a growing burden on health systems and require effective strategies to support long-term medication use. Medication Therapy Management (MTM) has been shown to improve clinical outcomes in chronic disease care, but its implementation depends on pharmacists’ competencies and access to structured training. In Indonesia, MTM remains inconsistently implemented in primary healthcare centers (PHCs), and pharmacists have reported competency gaps in the absence of standardized training. Evidence to inform context-specific training design is limited. The objective of this study was to explore and identify Indonesian PHC pharmacists’ perspectives on training for chronic-disease MTM, including their needs, preferences, perceived challenges, and facilitators.

**Methods:**

This paper reports the qualitative component of a larger mixed-methods study. A qualitative descriptive study was conducted using six focus group discussions (FGDs) with 32 PHC pharmacists from 32 provinces in Indonesia. Participants were purposively selected based on their experience in PHCs (≥ 6 months). All audio recordings were transcribed verbatim and then analyzed using an inductive thematic approach in Atlas.ti. To strengthen the credibility of the findings, we used triangulation, member checking, and peer debriefing during the analysis.

**Results:**

The analysis generated four themes: needs, preferences, challenges, and facilitators. Pharmacists emphasized a need for standardized, competency-based, and practice-oriented MTM training. They strongly preferred content that aligned with daily practice and focused on prevalent chronic diseases. However, they also identified several contextual challenges that might influence implementation, including geographical and cost barriers, limited digital access, and competing responsibilities. Institutional and policy support, as well as post-training mechanisms, were considered factors that may facilitate learning sustainability and implementation in routine care.

**Conclusions:**

Pharmacists in Indonesian PHCs perceive clear needs, preferences, challenges, and facilitators for MTM training. These findings can inform the development of context-sensitive, competency-based training programs. Embedding such training into continuing professional development frameworks is recommended to strengthen its sustainability and long-term impact, contributing to health professions education for primary care pharmacists.

**Supplementary Information:**

The online version contains supplementary material available at https://doi.org/10.1186/s12909-026-09722-6.

## Introduction

Globally, chronic diseases are the leading cause of mortality, responsible for 74% of all deaths, with most occurring in low- and middle-income countries (LMICs) [1, 2]. This growing burden requires strategies such as Medication Therapy Management (MTM), an approach designed to optimize medication use, improve adherence, and reduce medication-related problems [3, 4]. Its core elements include medication therapy review, personal medication record, medication action plan, intervention and referral, and documentation and follow-up, which reflect the expanding role of pharmacists in chronic disease care [3–5]. Through these elements, MTM enables pharmacists to act as integral members of the healthcare team by identifying and resolving medication-related issues and supporting patient self-management [3–5]. Recent systematic reviews and economic evaluations suggest that MTM improves outcomes for hypertension, diabetes, and dyslipidemia and is cost-effective in preventing cardiovascular complications [5–8].

In Indonesia, chronic diseases have become a major challenge for both the health system and economic development, particularly hypertension (30.8% among adults ≥ 18 years) and diabetes (11.7% among individuals ≥ 15 years), along with their risk factors [9–12]. Primary health care centers (PHCs) serve as frontline facilities responsible for early detection, management, and long-term monitoring of these conditions [13, 14]. To strengthen chronic disease management, the Indonesian government introduced MTM in 2017 as part of the Back-Referral Program (Program Rujuk Balik/PRB) [15–17]. The PRB facilitates continuity of care across settings: patients with chronic conditions may be referred to hospitals when unstable and referred back to PHCs once stabilized, with MTM services provided throughout this care pathway [15–17]. The program covers several prevalent diseases, including hypertension, diabetes, asthma, heart disease, and stroke [15]. Although MTM benefits for chronic disease care in PHCs have been reported [16–19], its delivery in practice remains inconsistent.

Pharmacists’ contributions are important for MTM success, as their competencies in pharmacotherapy, medication use assessment, and patient counseling support safe, effective, and patient-centered medication use [5, 20, 21]. However, a national survey of 1,132 PHC pharmacists in Indonesia found that although most had adequate knowledge, only about half demonstrated positive attitudes and practices toward MTM [4]. The survey also identified lack of interprofessional collaboration as a barrier to MTM implementation, reflecting both pharmacist‑related factors such as confidence and communication and system‑level factors like physician engagement and team workflow [4]. These findings reveal an educational gap in applied competencies that experience alone cannot address. Limited access to continuing professional education and standardized training further widens this gap [4, 22].

Addressing these issues requires strengthening pharmacists’ competencies through standardized and context-relevant training. Accordingly, this study is guided by the principles of competency-based education (CBE), which aligns training with practice needs, and continuing professional development (CPD), which emphasizes lifelong learning. A training needs assessment (TNA) is therefore necessary to systematically identify competency gaps and inform curriculum design [23].

Although TNA has been widely applied in healthcare education, evidence on MTM training needs among PHC pharmacists in Indonesia remains limited. No study has systematically addressed the educational gap: the absence of a standardized, practice-oriented MTM curriculum for primary care. Given the diversity of practice settings and regional contexts, understanding these needs at a national level is critical. Therefore, the objective of this study was to explore and identify Indonesian PHC pharmacists’ perspectives on training for chronic-disease MTM, including their needs, preferences, perceived challenges, and facilitators.

## Methods

This study adhered to the Consolidated Criteria for Reporting Qualitative Research (COREQ) [24] (see Table S1 in the Supplementary Materials).

### Study design

This paper reports the qualitative component of a larger mixed-methods study that included a national survey of PHC pharmacists. The quantitative survey results are reported elsewhere [4]. The same dataset was used in a parallel qualitative analysis that examined pharmacists’ pharmaceutical services at PHCs, focusing on the clinical pharmacy practice gap. The present analysis differs fundamentally: it addresses the educational gap and training needs, employs an independently developed coding framework, and has no thematic overlap with the parallel analysis.

A qualitative descriptive study was conducted using focus group discussions (FGDs) and inductive thematic analysis to explore pharmacists’ perspectives on training for chronic-disease MTM. A qualitative descriptive approach was chosen to provide a comprehensive summary of participants’ perspectives within real-world practice contexts and to inform the design and implementation of training programs [25]. FGDs were used to gather participants’ perspectives and to capture both individual experiences and shared professional practice across diverse PHC settings [26, 27]. The interactive nature of FGDs also facilitated collective reflection on how systemic and contextual factors shape pharmacists’ needs for MTM training.

### Study participants

We purposively recruited one pharmacist per province (34 provinces) through the Indonesian Pharmacists Association (IAI), the Public Health Pharmacy Association (Hisfarkesmas), and the Ciloto Health Training Center to capture diverse perspectives across Indonesia’s PHC system. PHC pharmacists were selected as the focus because they serve as the first point of contact in the healthcare system in Indonesia and face a less structured system for clinical pharmacy, making strengthening efforts more urgent than in hospital settings. Eligible participants were pharmacists who had worked in a PHC for at least six months and were involved in direct patient care; those not involved in direct patient care were excluded.

From the accessible pharmacists in each province, selection was guided by duration of PHC practice, practice setting (inpatient vs. outpatient), and the presence or absence of organizational roles in IAI or Hisfarkesmas to ensure variation in professional background rather than statistical representativeness. Recruiting one pharmacist per province aimed to achieve maximum variation across Indonesia’s decentralized PHC system rather than theoretical saturation within each province. A total of 32 pharmacists from 32 provinces participated. Two pharmacists from Papua and North Maluku were unable to attend due to limited internet connections.

### Ethical approval

This study received ethical approval from the Research Ethics Committee of Universitas Padjadjaran (No. 1096/UN6.KEP/EC/2023). Informed consent was obtained from all participants before data collection, including consent for audio and video recording. Participation was voluntary, and participants could decline to answer questions or withdraw at any time without consequence. Measures were taken to protect confidentiality throughout the study. As a token of appreciation, participants received an incentive and two professional credit units from the IAI.

### Topic guide development

We developed a semi-structured topic guide to explore pharmacists’ experiences in managing chronic disease patients and their perspectives on MTM training. The guide was prepared in Bahasa Indonesia by FR (a doctoral student with qualitative research training and experience) and SDA (a senior qualitative researcher). The topic guide was developed based on the study objectives and informed by the methodological framework of Kallio et al. (2016) for developing qualitative semi-structured interview guides [28]. Questions were designed to cover the key domains of a training needs assessment: current competencies, desired training content and delivery, and perceived barriers and facilitators. IMP reviewed the guide, and it was pilot-tested with two pharmacists. We then made minor changes to improve clarity and flow.

The guide covered key areas such as pharmacists’ practice experiences, prior training, perceived importance of MTM training, preferred training approaches, and contextual challenges. Example questions included: “How important is MTM training for chronic disease management, and how should it be delivered?” (see Table S2 in the Supplementary Materials). Additional prompts were used when needed to clarify and expand participants’ responses. All discussions were conducted in Bahasa Indonesia; therefore, no translation of the guide was required.

### Data collection procedure

From 19 to 21 October 2023, FGDs were conducted via Zoom (Zoom Communications, Inc., San Jose, United States) with 32 PHC pharmacists from 32 provinces in Indonesia. The online format was chosen to enable participation across geographically dispersed regions, with the focus placed on discussion content over non-verbal cues [29]. Six groups of four to six pharmacists were formed based on time zones and availability to allow effective discussion [30, 31]; the group format allowed participants to compare experiences across diverse PHC settings, enriching the thematic analysis. Informed consent and sociodemographic data, including the frequency of clinical pharmacy services performed, were collected before the FGDs.

Each session lasted up to two hours and was facilitated by FR (a female doctoral student trained in qualitative research), whose interest in chronic disease MTM was disclosed to participants. The sessions were supported by three team members (MH: host; TN: note-taker; AMU: research assistant). No prior relationship was established with the participants. During discussions, quieter participants were encouraged to share their views, and more dominant voices were gently moderated. At the end of each session, the main points were summarized and checked with participants [32]. Given the aim was to capture maximum variation across Indonesia’s diverse and decentralized PHC system, we prioritized breadth of perspectives from all 32 provinces rather than thematic saturation. This approach is consistent with qualitative descriptive studies where the goal is to describe a phenomenon across heterogeneous contexts rather than to develop theory [33, 34]. Although thematic saturation was not the primary aim, recurring patterns were consistently observed across all six FGDs, indicating sufficient information power to support thematic development while preserving contextual diversity.

### Data analysis

FGD recordings were transcribed verbatim by FR and independently coded by FR and TN. An open coding approach was used [35]. We read the transcripts several times and then assigned initial codes to relevant segments. We used sentences rather than isolated words as units of analysis to preserve contextual meaning. Coding results were compared and refined through discussion, with disagreements resolved by consensus or consultation with other researchers (SDA and IMP).

The data analysis followed a thematic analysis approach, in which the final set of codes was organized into categories, subthemes, and overarching themes [36]. Atlas.ti (Lumivero, Berlin, Germany) was used to manage the data. We used an inductive approach to allow themes to emerge from the data without imposing a predefined framework [35, 37, 38]. This approach was considered appropriate given the limited existing research on MTM training in Indonesia while enabling the identification of context-specific insights. Themes and subthemes were named in relation to the research questions and subsequently refined in multiple rounds to ensure they accurately represented participants’ perspectives and were clearly distinguishable from one another [38, 39].

Coding was performed across all six FGDs, and themes were developed from the entire dataset, ensuring that findings reflect the combined perspectives of pharmacists from all 32 provinces rather than any single group or region. The research team was involved from transcription to interpretation, with regular verification and debriefing during the analysis.

All FGDs were conducted and analyzed in Bahasa Indonesia to keep the original meaning. Excerpts included in this paper were translated into English by FR and reviewed by co-authors to ensure accuracy and consistency of meaning.

### Data validation

We conducted a peer debriefing with three researchers (SDA, TN, and IMP) to review and refine the interpretation of the findings. In addition, a summary of the findings was shared with selected participants representing different regions and levels of engagement in the FGDs. Participants were invited to give feedback using guided questions to check whether the interpretations were clear and relevant [40]. This process enhanced the credibility and accuracy of the study findings [41].

### Data quality control

Several measures were applied to ensure the study’s trustworthiness. FGD transcripts were checked for accuracy by the research team and returned to selected participants for verification. Data triangulation was conducted by examining relevant documents, including regulations, guidelines, reports, and previous studies, to support and contextualize participants’ accounts [42, 43].

To minimize potential researcher bias, FR practiced reflexivity by keeping reflective notes and using bracketing during the analysis. Detailed note-taking, independent coding by FR and TN, and regular discussions with the research team further reduced single-researcher influence and strengthened analytical consistency. Documentation of participants’ characteristics supported transferability.

## Results

### Respondents’ characteristics

Six FGDs were conducted with 32 pharmacists from PHCs across 32 provinces in Indonesia. Most participants were female, with a mean age of 35.6 years, and the majority held a professional pharmacy degree. Over half had graduated more than 10 years ago, and nearly half had worked in PHCs for more than five years. Participants were almost evenly divided between inpatient and outpatient settings.

In most PHCs, only one pharmacist was available, often supported by one or two pharmacy technicians. Most participants had attended general training on pharmaceutical services, either clinical or logistical, but few had completed the standardized program specifically for PHC pharmacy personnel. These data provide a baseline of prior training exposure, as no formal MTM training has yet been available for PHC pharmacists in Indonesia. Participants’ demographic characteristics are summarized in Table 1.

**Table 1:** Participants’ demographic characteristics (*n* = 32)

Characteristics	*n*	%
Sex		
Male	8	25.00
Female	24	75.00
Age (mean ± SD)	35.6 ± 5.9	
Educational background		
Pharmacist professional	27	84.38
Master	5	15.62
Years since graduating from professional education		
1–5	7	21.88
6–10	8	25.00
> 10	17	53.12
Duration of practice in PHC		
Six months to one year	1	3.12
More than one year to five years	16	50.00
More than five years	15	46.88
Practice settings		
Inpatient	15	46.88
Outpatient	17	53.12
The number of pharmacists in the PHC		
Only one	21	65.62
Two	8	25.00
More than two	3	9.38
The number of pharmacy technicians in PHC		
None	3	9.38
One	10	31.25
Two	11	34.38
More than two	8	25.00
Have you ever attended training to support pharmaceutical service delivery*?		
Yes	26	81.25
No	6	18.75

*Training refers to general training related to pharmaceutical service delivery (clinical or logistical). No standardized MTM training has yet been conducted for PHC pharmacists in Indonesia

**Fig. 1:**
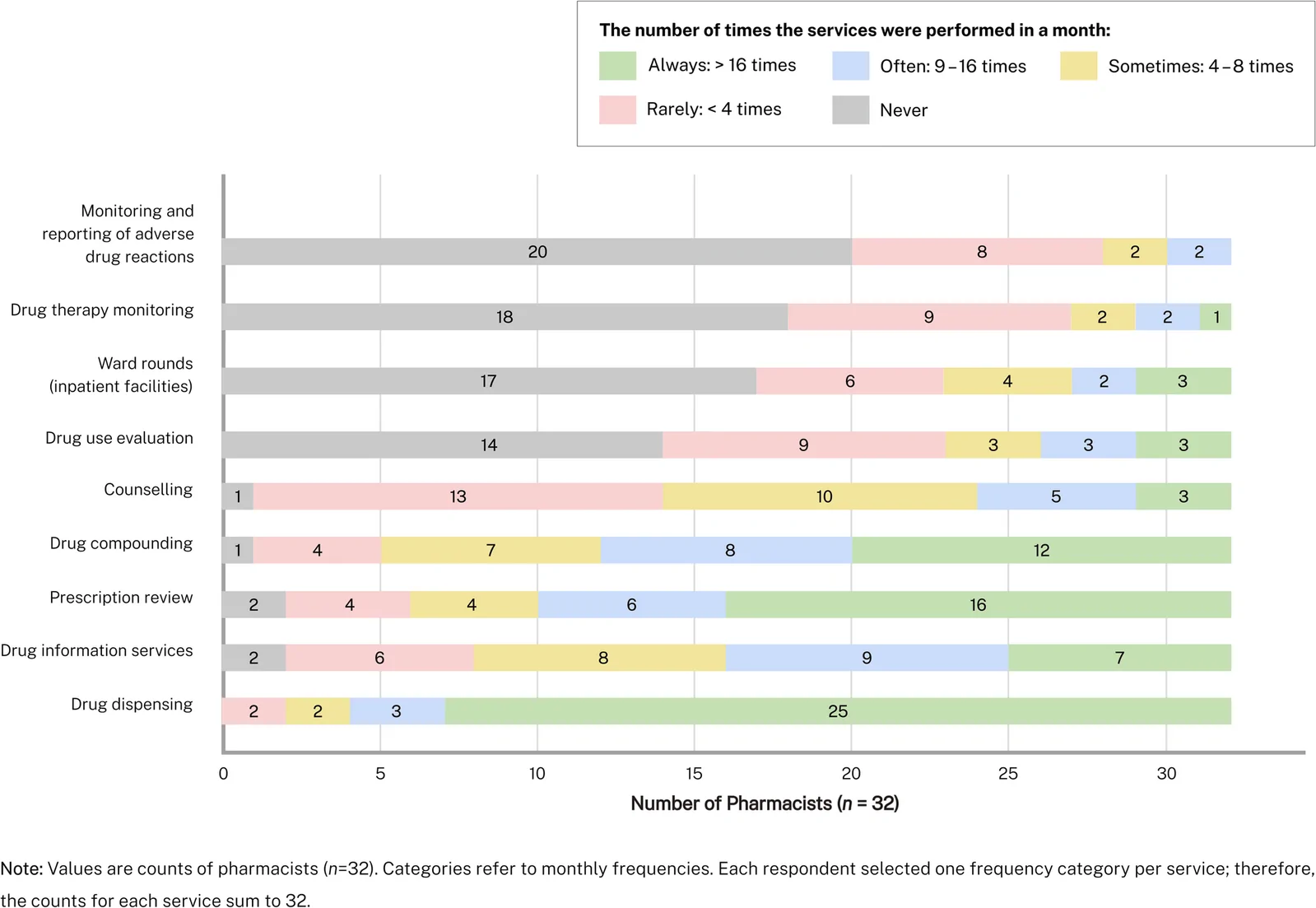
The frequency of clinical pharmacy services performed by participants in a month

Figure 1 shows how often participants performed clinical pharmacy services outlined in the Technical Guidelines for Pharmaceutical Service Standards at PHCs [44]. For each service, participants selected a single frequency category; therefore, the counts for each service sum to 32. Prescription review and drug dispensing were performed > 16 times per month by 16 and 25 respondents, respectively, indicating these services were routine for half to most of the sample. Other activities, including side effects monitoring, ward rounds (in inpatient facilities), and drug therapy monitoring, were reported less frequently.

### The generation of themes

Through open coding, 30 codes were identified and grouped into eight subthemes, which were then organized into four themes: Needs for MTM Training, Preferences for MTM Training, Challenges in MTM Training Implementation, and Facilitators for MTM Training. These themes represent key dimensions of training needs identified in the data. The findings were reviewed with selected participants as part of the trustworthiness process. Participant identifiers are presented as (Pxx, practice setting, years of experience in PHCs).

**Fig. 2:**
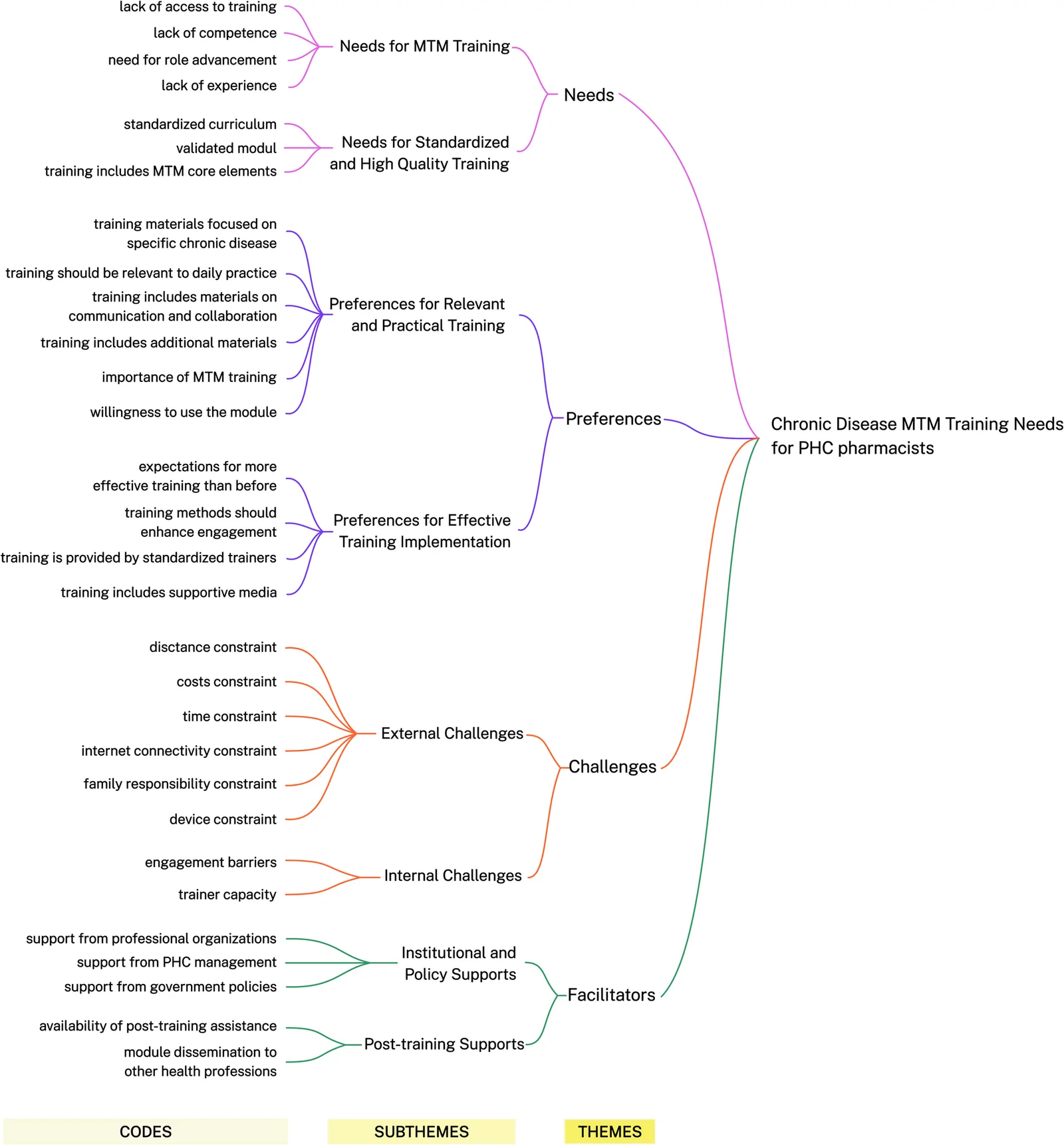
The coding tree

Figure 2 shows the relationships between themes, subthemes, and codes, while Table 2 summarizes the themes and subthemes with brief descriptions. A more detailed coding framework, including all codes and the number of excerpts supporting each code, is provided in the Supplementary Materials (Table S3). The number of excerpts reflects topics more frequently raised in participants’ narratives rather than quantitative prevalence. Across the FGDs, the most frequently raised topics included the need for MTM core elements, limited training access, and lack of competence. Training materials on specific chronic diseases, such as hypertension and diabetes, were also frequently highlighted. Perspectives differed on disease priorities beyond hypertension and diabetes, as well as on joint training with other healthcare professionals.

**Table 2:** Summary of themes and subthemes emerged from FGDs

Theme	Subtheme	Subtheme description
Needs	Needs for MTM Training	Pharmacists’ needs for MTM training to improve their competencies and roles in clinical pharmacy services.
	Needs for Standardized and High-Quality Training	The need for a training curriculum that is nationally standardized, validated, and consistently high in quality.
Preferences	Preferences for Relevant and Practical Training	Pharmacists’ preferences for training content that is directly applicable to daily practice and relevant to their work context.
	Preferences for Effective Training Implementation	Pharmacists’ preferences for how the training should be delivered, including methods, formats, and support mechanisms.
Challenges	External Challenges	Obstacles to training participation and implementation that arise from factors outside the control of training organizers.
	Internal Challenges	Obstacles related to the training design, delivery process, and trainer capacity.
Facilitators	Institutional and Policy Supports	Forms of support from professional organizations, PHC management, and government policies that enable MTM training.
	Post-training Supports	The need for ongoing assistance, peer learning platforms, and the dissemination of materials after the training is completed.

### Theme 1: needs

This theme included two subthemes: needs for MTM training and for standardized, high-quality training. It reflects pharmacists’ main training needs for MTM, shaped by limited access to relevant training, gaps in clinical competence, and few opportunities to expand their roles. It also points to the need for a standardized curriculum and validated modules.

#### Subtheme 1.1: needs for MTM training

Training opportunities tailored for PHC pharmacists were rare. One participant noted,

“At the PHC, we rarely receive training that updates our knowledge.” (P3, outpatient, > 5 years).

Even when training was available, it was often not relevant to MTM or clinical practice and focused more on logistics:

“Most pharmacy-related meetings or training at PHCs focus more on logistics, rarely covering clinical pharmacy services.” (P10, outpatient, 1–5 years).

Gaps in knowledge and confidence limited clinical practice:

“Pharmaceutical services are often limited due to resource constraints and lack of knowledge.” (P32, inpatient, 1–5 years).

Many participants felt their roles remained administrative:

“We have 15 PHCs, and only a few pharmacists are involved in clinical services. The rest manage the warehouse.” (P24, inpatient, 1–5 years).

Participants believed MTM training could enhance their competencies and advance their roles in clinical pharmacy services.

“With MTM training, perhaps the focus of pharmacists' work could shift from management to clinical pharmacy.” (P8, outpatient, > 5 years).

#### Subtheme 1.2: needs for standardized and high-quality training

Pharmacists stressed the need for standardized, high-quality training to improve clinical pharmacy services. The need for a standardized curriculum was noted to ensure consistency across regions:

“When standardized, the curriculum is practical … from Sabang to Merauke, it’s exactly the same.” (P3, outpatient, > 5 years).

Validated modules were also considered important for alignment with clinical pharmacy standards and for supporting interprofessional credibility:

“If we bring a validated module recognized by various healthcare professions, we have a strong foundation.” (P16, inpatient, 1–5 years).

High-quality training was expected to include core MTM elements, such as personal medication records to track patients’ use of outside medications or herbs:

“… we don’t know if patients buy other drugs outside, consume herbs, or visit other places, so there’s no intervention taken.” (P28, inpatient, 1–5 years).

### Theme 2: preference

This theme comprises preferences for relevant and practical training and for effective training implementation. Training was expected to be applicable in daily practice, focus on common chronic diseases, and include practical components.

#### Subtheme 2.1: preference for relevant and practical training

Participants preferred training applicable to daily practice, focusing on prevalent chronic diseases such as hypertension and diabetes.

“First, we look at the most prevalent chronic diseases, for example, hypertension.” (P21, inpatient, > 5 years).

While these diseases were most frequently prioritized, some participants suggested including locally relevant conditions, such as tuberculosis, HIV, and hyperlipidemia.

Practical content was valued, including case studies aligned with PHC formularies and real‑world examples from experienced practitioners:

“Practitioners often provide relatable examples … it is easier to connect …” (P29, outpatient, < 1 year).

Views on interprofessional training were mixed; while some supported joint sessions, others preferred pharmacist‑focused training:

“Since it’s designed for pharmacists, the focus should remain on them.” (P11, outpatient, > 5 years).

Participants also emphasized skills in communication and collaboration:

“If we are equipped with how to collaborate well … we can provide coordinated professional care and solve patient issues together.” (P30, outpatient, > 5 years).

Additional suggested content included drug interactions, herbal medicine, cost-effectiveness, and digital skills.

Pharmacists recognized that MTM training is relevant to their current competency gaps and daily practice challenges. Overall, there was strong interest in using MTM training materials when relevant:

“If the module is available, I will be very willing to use it.” (P14, outpatient, 1–5 years).

#### Subtheme 2.2: preference for effective training implementation

Face‑to‑face training was preferred over online formats because it allowed better engagement:

“… it would be better to have it in person.” (P6, outpatient, > 5 years).

Interactive, hands-on approaches were valued, as

“… theory is easy to forget.” (P25, inpatient, > 5 years).

Some participants also recognized the value of digital support tools, such as instructional videos, to reinforce learning and support continued practice (P28, inpatient, 1–5 years). In addition, standardized trainers were identified as important for consistent knowledge transfer:

“Both tutors and training materials received by the participants will be consistent.” (P1, inpatient, > 5 years).

### Theme 3: challenges

This theme comprises external (beyond training organizers’ control) and internal challenges (related to training design and delivery).

#### Subtheme 3.1: external challenges

For face‑to‑face training, pharmacists in remote areas faced travel distance, cost, and family responsibilities:

“If we travel to Java, it’s a very long journey.” (P17, outpatient, > 5 years).

“But if it’s offline, … I had to bring my child.” (P24, inpatient, 1–5 years).

Online formats introduced different barriers, such as poor internet connectivity and device issues:

“There are remote areas in my region … there’s no signal.” (P26, outpatient, > 5 years).

#### Subtheme 3.2: internal challenges

Online training was often described as difficult to follow due to competing work or home demands:

“Online training often overlaps with work, making it hard to focus.” (P22, outpatient, > 5 years).

Some participants suggested that in-person, small group workshops could improve engagement:

“… possibly as a workshop in small groups of about 6–8 people to maintain focus.” (P8, outpatient, > 5 years).

Views on trainer capacity were mixed: some trusted central trainers, while others felt they might lack local practice knowledge.

“Perhaps central trainers view things from only one perspective, a general point of view.” (P23, inpatient, 1–5 years).

### Theme 4: facilitators

This theme includes professional organizations, PHC management, and government policies, as well as post-training support.

#### Subtheme 4.1: institutional and policy support

Professional organizations were seen as key to promoting standardized training and attracting participants:

“They [professional organizations] will also help promote the module and attract participants.” (P19, inpatient, 1–5 years).

Support from PHC management was also important but not always easy to obtain.

“There is the challenge of obtaining permission from the head of the PHC.” (P26, outpatient, > 5 years).

Government policy was also considered important for legitimizing training and encouraging participation:

“. . especially validated and approved by the policymakers.” (P10, outpatient, 1–5 years).

#### Subtheme 4.2: post-training support

Participants highlighted the importance of post-training support to sustain learning and support implementation in practice. They valued peer support through platforms like WhatsApp groups for sharing difficulties:

“There’s a WhatsApp group … a place to ask questions.” (P24, inpatient, 1–5 years).

“Because with a WhatsApp group, we can share our difficulties.” (P28, inpatient, 1–5 years).

Broader dissemination of training materials across health professions was also mentioned to support shared understanding and collaboration:

“Other health professions should also know about it [the module].” (P19, inpatient, 1–5 years).

### Data triangulation: document analysis

Triangulation results aligned with FGD findings (Table 3). Previous studies confirmed gaps in pharmacist training and competencies, with pharmacists often focused on administrative rather than clinical tasks [4, 45, 46]. Government regulations (e.g., Government Regulation No. 67 of 2019) and curriculum guidelines emphasized the need for standardized training and validated modules, supporting participants’ views [47, 48]. Policies also highlighted hypertension and diabetes as priority conditions for chronic disease management in PHCs [9, 49, 50]. The importance of interprofessional collaboration skills was echoed in prior research [4, 46]. Overall, triangulation reinforced the consistency of findings across data sources.

**Table 3:** Findings from document analysis

Theme	Source	Key findings	Consistency with FGD results
Needs	Research article [46]	Significant gaps in pharmacy workforce development, especially in competency development and early-career training, underscoring the lack of systematic training for pharmacists, including those in PHCs.	Consistent: PHC pharmacists reported limited training access and a lack of standardized curriculum.
	Research article [4]	Knowledge gaps in MTM elements, as well as limited attitudes and skills for MTM implementation.	Consistent: pharmacists felt unprepared and less competent in clinical pharmacy services, including MTM.
	Research article [45]	The majority (96.7%) of PHC pharmacists focus on medication logistics over clinical pharmacy services.	Consistent: FGD shows pharmacists’ roles are more centered on logistics than on clinical services.
	Government Regulation [48]	Training programs must meet professional and competency standards to ensure quality and effectiveness.	Consistent: Participants stressed a standardized curriculum to improve competencies and service quality in PHCs.
	Guidelines [47]	Standardized curriculum and modules are important for effective competency development.	Consistent: FGD findings highlight the critical role of standardized curriculum and validated modules for effective competency improvement.
Preferences	Regulation [49]	Hypertension is a primary chronic disease focus in Indonesia.	Consistent: FGD suggests that training content should address pressing health challenges like hypertension.
	Regulation [50]	The Prolanis program focuses on managing hypertension and diabetes in PHCs.	Consistent: pharmacists stressed the need for MTM training targeting chronic diseases relevant to the Prolanis program.
	Report [9]	Hypertension is the most prevalent chronic disease (29.2%–30.8% of adults based on blood pressure measurements).	Consistent: pharmacists reported that hypertension is the most prevalent chronic disease; thus, the training needs to be focused on the disease.
	Research article [4]	PHC pharmacists perceived a lack of interprofessional collaboration and patient communication as major barriers to MTM implementation. Thus, training should address these issues.	Consistent: pharmacists believed that training should enhance collaboration and communication skills for PHC pharmacists.
	Research article [46]	Policy gap in fostering collaboration among healthcare professionals. Training should address team-based working skills.	Consistent: FGD suggests that collaboration is essential and should be included in structured training programs like MTM training.
	Research article [4]	The majority (92.6%) of PHC pharmacists were interested in training. They also perceived that the lack of training was a barrier to MTM implementation.	Consistent: both highlight MTM training as critical to improving the competency of PHC pharmacists, especially for chronic disease care.

## Discussion

This study provides national-level evidence to inform the design of MTM training for pharmacists in Indonesian PHCs. The findings clarify not only what competencies need to be strengthened, but also how training should be delivered and what conditions are required to support its implementation in routine practice.

Pharmacists described gaps in clinical competencies, especially in MTM-related skills (e.g., medication review, patient counseling, therapy monitoring), which persisted even among experienced participants. This finding indicates that experience alone is insufficient without structured, ongoing training. Pharmacists also reported limited access to clinically oriented training. Existing programs focus on logistics rather than clinical decision-making, reflecting a historical emphasis on pharmaceutical management that has shaped both training priorities and professional identity in Indonesian PHCs [45, 46, 51]. Similar patterns appear in other LMICs, where primary care pharmacists lack opportunities for clinical training [52]. This focus on logistics stems from a system that measures pharmacy performance through managerial rather than patient‑centered outcomes [53]. Consequently, many pharmacists lacked practical MTM experience, trapped in a cycle of low confidence and limited clinical engagement. Addressing this gap requires both clinical skills training and an institutional shift in role expectations.

Structured MTM training can catalyze this shift by equipping pharmacists with competencies and evidence to advocate for an expanded clinical role. However, such training must be built on a nationally standardized curriculum and validated modules recognized across health professions. The absence of such standardization has led to fragmented, locally initiated workshops lacking a unified competency framework and quality assurance. A nationally standardized curriculum covering all five core MTM elements would provide a consistent, evidence‑based foundation, legitimizing pharmacists’ clinical role, establishing a clear career pathway, and reinforcing the needed institutional shift.

Pharmacists strongly preferred training that is directly applicable to daily practice and delivered interactively, focusing on the most prevalent chronic diseases to maximize immediate patient impact. They preferred practice‑oriented and interactive approaches, which support knowledge retention and skill application more effectively than didactic formats [54]. Participants also valued short theoretical sessions, suggesting that combining approaches may be useful. Others suggested additional topics, such as herbal medicines and cost‑effectiveness, indicating a desire for a well‑rounded skill set beyond core priorities. Pharmacists also called for standardized trainers to ensure consistent quality across regions, addressing the current variability in training delivery.

Besides MTM core elements, communication and collaboration skills were consistently highlighted, though participants held different views on how collaboration materials should be integrated. We infer that these views reflect varying levels of existing interprofessional collaboration [55–58]. Pharmacists accustomed to working with physicians may welcome joint sessions, whereas those in more siloed environments may first need profession‑specific competency building. Hence, training should be incremental: start with pharmacist‑focused modules, then progressively add interprofessional joint sessions.

Pharmacists identified barriers rooted in Indonesia’s decentralized geography and unequal digital infrastructure. These barriers disproportionately affect remote areas and women with dual work-home responsibilities. Internal challenges included unengaging training formats and variability in trainer expertise. These external and internal challenges were closely linked to preferences for face‑to‑face versus online formats. The two are complementary: face‑to‑face workshops provide interactivity and hands‑on practice, while online components offer practical reinforcement across distant regions. A blended learning approach combining short in‑person workshops with self‑paced online modules and virtual case discussions [59–61] offers the most feasible compromise. In addition, training‑of‑trainers models, supported by standardization mechanisms, can address skepticism about trainers’ capacity while maintaining local relevance [26, 62, 63].

Workforce constraints should also be considered in designing the training. In many PHCs, pharmacists work with limited personnel and manage competing responsibilities, restricting both training attendance and time to apply new skills. To be feasible, training should be modular [64], allowing incremental implementation. A concrete approach would be to start MTM with PRB patients with hypertension or diabetes, use a brief, standardized MTM documentation template, and delegate routine dispensing to pharmacy technicians during protected clinical time [65, 66].

This study underscores the importance of system-level support for training sustainability. Participants identified institutional backing, policy alignment, and professional organization collaboration as key enablers because MTM is not yet a formal priority in Indonesian PHCs and participation often depends on individual initiative. Post-training, pharmacists valued peer networks (e.g., WhatsApp groups) to apply newly acquired skills and reduce isolation, especially for those working alone. Evidence from India, Nepal, and Nigeria confirms that virtual follow‑up can improve provider performance [67]. Broader dissemination of MTM modules to other healthcare professionals was also recommended to enhance team-based care [68].

Successful MTM training therefore requires a dual support system: (1) structural enablers such as policy backing, professional organization endorsement, and integration into the national CPD credit system to encourage participation; and (2) sustained post-training mechanisms to foster a collaborative environment for clinical role expansion. Structured outcome evaluation using frameworks like the Kirkpatrick model would ensure measurable improvements in pharmacist performance and patient outcomes [69].

Although an inductive thematic approach was employed, the study findings align with established educational theories. Practice‑oriented, disease‑specific content reflects competency‑based education (CBE), which advocates for curricula designed around actual practitioner tasks [70]. Preference for interactive, case‑based learning resonates with experiential learning theory, where knowledge is constructed through concrete experience and reflection [71]. The need for post‑training support and system‑level backing mirrors effective CPD models, which stress that sustained practice change requires ongoing reinforcement [72]. By situating these inductively derived findings within recognized frameworks, this study bridges health professions education and health systems strengthening, offering evidence to inform both curriculum design and the integration of pharmacists into chronic disease care teams in primary care. Taken together, the four themes define a context‑sensitive CPD model for MTM that integrates a competency‑based curriculum, blended delivery, institutional endorsement, post‑training support, and outcome evaluation. This model may serve as a template for similar initiatives in other LMICs.

### Study strengths, limitations, and implications

This study has several strengths. The involvement of pharmacists from PHCs across multiple provinces in Indonesia captures a range of perspectives relevant to national-level training design. Differences in practice settings, experience, and regional contexts allowed a more detailed understanding of MTM training needs across primary care environments. Several steps were taken to strengthen methodological rigor, including triangulation, peer debriefing, iterative theme development, and member checking.

Several limitations should be noted. The data relied on self-reports and did not include direct observation, which may introduce social desirability bias and limit insight into actual practice. We used rapport building, careful questioning, cross-group comparison, and triangulation to reduce these issues [73]. The study did not involve other stakeholders (e.g., patients, policymakers, other healthcare professionals) whose views may further inform the training design. Recruitment through professional organizations may also have influenced participation, although we aimed to include pharmacists with and without organizational roles. Thus, the competency gaps and training needs reported likely represent conservative estimates; less engaged pharmacists might have even greater gaps. However, although the study emphasized maximum variation sampling rather than thematic saturation, recurring themes were consistently observed across all six FGDs, suggesting adequate information power to support thematic development. In addition, online FGDs posed challenges (digital access, limited non-verbal interaction) that may have hindered effective communication and engagement, which were addressed through smaller group sizes and active facilitation during discussion. The absence of participants from Papua and North Maluku, due to connectivity issues, may slightly limit the transferability of findings to these regions. Additionally, while participants represented diverse settings and experience levels, the sample size per subgroup was too small for formal comparative analysis.

Further research is needed to design and evaluate structured MTM training programs for PHC pharmacists, particularly on how such programs improve competency and service delivery. Longitudinal studies could assess the sustainability of training outcomes. Future studies should also incorporate mixed methods and perspectives from multiple stakeholders. Larger, stratified samples could examine how training needs differ across characteristics.

This study has important implications for MTM training development in primary care. Building on the findings, we propose a concrete training model for Indonesian PHCs:

1. The MTM curriculum should be competency‑based, initially focused on hypertension and diabetes, with subsequent modules for other chronic diseases.
2. Delivery should adopt a blended format. For example, starting with online self‑paced modules for foundational knowledge, followed by in‑person workshops for case‑based learning, role‑play, simulations, and hands‑on practice.
3. Post‑training support should leverage existing WhatsApp groups for peer consultation, plus periodic refresher sessions.
4. A training‑of‑trainers approach is recommended: master trainers standardize materials, while regional facilitators deliver training to ensure local relevance.
5. The program should be embedded into the Ministry of Health’s national CPD credit system for pharmacists. Completion of the MTM training modules would provide CPD credits required for license renewal, creating a structural incentive for sustained participation and supporting long-term program viability.
6. Training evaluation should follow an established framework, such as the Kirkpatrick model, which assesses outcomes at four levels: participant reactions, learning gains, behavioral changes in practice, and patient-level outcomes [69].

This structured pathway, supported by institutional backing, policy alignment, and professional organization collaboration, ensures MTM training is part of a sustained professional development system with lasting impact on chronic disease care.

## Conclusions

This study shows that pharmacists in Indonesian PHCs perceive a clear need for MTM training to strengthen applied clinical competencies. They prefer practice-oriented training, focused on common chronic conditions, and delivered through interactive and relevant learning approaches. At the same time, participation and implementation are influenced by contextual challenges, including logistical constraints, limited digital access, and competing responsibilities. Facilitators such as institutional support, policy alignment, and post-training mechanisms are considered important to sustain learning and support practice change. These findings can inform the development of context-sensitive, competency-based training programs for pharmacists in primary care. Embedding such training into continuing professional development frameworks is recommended to strengthen its sustainability and long-term impact on chronic disease care. This study contributes to health professions education by providing a national training needs assessment that can guide the development of MTM curricula for primary care pharmacists in Indonesia and similar LMIC settings.

## Supplementary Information


Supplementary Material 1.



Supplementary Material 2.



Supplementary Material 3.


## Data Availability

A subset of qualitative data from the focus group discussions is presented in this article, and the full data set is available from the corresponding author upon reasonable request.

## Abbreviations

COREQ: Consolidated Criteria for Reporting Qualitative Research
FGD: Focus Group Discussion
Hisfarkesmas: Public Health Pharmacy Association (Himpunan Seminat Farmasi Kesehatan Masyarakat)
IAI: Indonesian Pharmacists Association (Ikatan Apoteker Indonesia)
LMICs: Low- and Middle-Income Countries
MTM: Medication Therapy Management
PHC: Primary Healthcare Center
PRB: Back-Referral Program (Program Rujuk Balik)
Prolanis: Community-Based Chronic Disease Management Program (Program Pengelolaan Penyakit Kronis)
TNA: Training Needs Assessment
