# Supplementary material for: Pharmacists’ perspectives on medication therapy management training needs for chronic disease care in Indonesian primary healthcare centers: a qualitative study across 32 provinces
Source: BMC Med Educ. 2026 Jun 19;26:1402. doi: 10.1186/s12909-026-09722-6 (PMC13527914; doi:10.1186/s12909-026-09722-6)
Supplement: Supplementary file 2 [file 12909_2026_9722_MOESM2_ESM.pdf]

Table S2 Topic guide

Topic
**Overview of the Actual Practice of Pharmaceutical Services for Chronic Disease Patients and Pharmacists' Perceptions of Their Role in These Services**
1. Tell us about your experience in providing pharmaceutical services for chronic disease patients.
2. What is your view about your role as a pharmacist in pharmaceutical services for chronic disease patients?
**Pharmacist's View on the Content, Benefits, and Limitations of Existing Training and Its Delivery**
1. 1. Have you ever attended training that supports pharmaceutical services at the PHC? Can you also tell us the name of the training and when did you follow it?<br>2. What do you think about the training regarding content relevance (according to practical needs), benefits (e.g., improving competence), whether it can be applied, and its limitations?
2. Tell us about the delivery of the training, for example, by whom, where, in what format, and the advantages and disadvantages of the training delivery.
**Overview of Chronic Disease MTM Training Needs: Needs/Benefits, Implementation, Disease Focus, and Resource Constraints/Challenges**
1. How important is the Chronic Disease MTM training to be held? How should it be organised, or what method is appropriate?
2. Do you think the training should focus on specific chronic diseases? Please explain why! And if yes, what chronic disease should be focused on and why?
3. What do you think about the constraints or challenges of resources (number of human resources, costs, or infrastructure) in implementing the training? What is your advice to overcome these obstacles?
4. Do you think it is necessary to standardise the MTM training curriculum for all pharmacists in Indonesia? Explain why!
5. How important is it to validate the module if it can be used as technical guidance or guidance in providing pharmaceutical services for chronic disease patients? Explain why!
6. How important do you think the following topic is to be the content of the MTM training module?<br>• First, Medication Therapy Review. It may include a Comprehensive (annual review) and Targeted Review (when medication problems occur).<br>• Second is the patient's medication record (including prescription, non-prescription, supplement, and herbal medications). This topic can be how to create/record and evaluate the record.

	• Third, a Medication-Related Action Plan. It may be related to designing strategies to prioritise drug-related problems, making plans to overcome these problems, and how to implement them.<br>• Next, Intervention and Referral. It could involve techniques in providing consultation or counselling services and intervening to overcome drug-related problems.<br>• Fifth, Documentation and Follow-up, including documentation techniques, such as Patient Treatment Records, Follow-up Plans, or SOAP records (Subjective, Objective, Assessment, Plan), both for internal, patient, and third parties such as BPJS.<br>• Then, what do you think about communication with patients and interprofessional collaboration being included in the MTM training module?
7.	If the MTM training module is available, will you use the module? Explain why!
8.	Do you have any ideas or suggestions that we must consider in organising Chronic Disease MTM training?
