# Supplementary material for: Pharmacists’ perspectives on medication therapy management training needs for chronic disease care in Indonesian primary healthcare centers: a qualitative study across 32 provinces
Source: BMC Med Educ. 2026 Jun 19;26:1402. doi: 10.1186/s12909-026-09722-6 (PMC13527914; doi:10.1186/s12909-026-09722-6)
Supplement: Supplementary file 3 [file 12909_2026_9722_MOESM3_ESM.pdf]

Table S3 Detailed coding framework: themes, subthemes, codes, and number of excerpts

Theme	Subtheme	Codes and subcodes	Number of excerpts (*N*)
Needs	Needs for MTM Training	1. Lack of Access to Training	63
		2. Lack of Competence	54
		3. Need for Role Advancement	24
		4. Lack of Experience	1
	Needs for Standardized and High-Quality Training	1. Standardized Curriculum	30
		2. Validated Module	35
		3. Training Includes MTM Core Elements	111
		3.1 Medication Therapy Review	25
		3.2 Personal Medication Record	28
		3.3 Medication Action Plan	22
		3.4 Intervention and Referral	19
		3.5 Documentation and Follow-up	17
Preferences	Preferences for Relevant and Practical Training	1. Training Materials Focused on Specific Chronic Diseases	43
		1.1 Hypertension	23
		1.2 Diabetes	23
		1.3 Tuberculosis	6
		1.4 HIV	2
		1.5 Hyperlipidemia	2
		1.6 Hypercholesterolemia	1
		1.7 Heart Disease	1
		2. Training Should be Relevant to Daily Practice	33
		2.1 Training Involves Experienced Experts	15
		2.2 The Presence of a Role Model	7
		2.3 Training Involves Interprofessional Session	6
		2.4 The Use of Case-based Learning	3
		2.5 Training Materials Adjusted with the Relevant Formularium in PHC	2
		3. Training Includes Materials on Communication and Collaboration	32

Theme	Subtheme	Codes and subcodes	Number of excerpts (*N*)
		4. Training Includes Additional Materials	16
		5. Importance of MTM Training	36
		6. Willingness to Use the Module	21
	Preferences for Effective Training Implementation	1. Expectations for More Effective Training Than Before	21
		2. Training Methods Should Enhance Engagement	22
		3. Training is Provided by Standardized Trainers	6
		4. Training Includes Supportive Media	7
Challenges	External Challenges	1. Distance Constraint	19
		2. Costs Constraint	15
		3. Time Constraint	11
		4. Internet Connectivity Constraint	8
		5. Family Responsibility Constraint	3
		6. Device Constraint	1
	Internal Challenges	1. Engagement Barriers	28
		2. Trainer Capacity	6
Facilitators	Institutional and Policy Supports	1. Support from Professional Organizations	8
		2. Support from PHC Management	4
		3. Support from Government Policies	2
	Post-training Supports	1. Availability of Post-training Assistance	5
		2. Module Dissemination to Other Health Professions	3

Note: The number of excerpts indicates how often a code appeared across participants' narratives. These counts are presented for descriptive purposes only, to give a sense of which topics received more or less emphasis in the discussions, and are not intended as measures of statistical significance or generalizability.
